# Single-pass Kelvin force microscopy and d*C*/d*Z* measurements in the intermittent contact: applications to polymer materials

**DOI:** 10.3762/bjnano.2.2

**Published:** 2011-01-06

**Authors:** Sergei Magonov, John Alexander

**Affiliations:** 1 Agilent Technologies, 4330 Chandler Blvd., Chandler, AZ 85226, U.S.A.

**Keywords:** atomic force microscopy, fluoroalkanes, Kelvin force microscopy, surface potential

## Abstract

We demonstrate that single-pass Kelvin force microscopy (KFM) and capacitance gradient (d*C*/d*Z*) measurements with force gradient detection of tip–sample electrostatic interactions can be performed in the intermittent contact regime in different environments. Such combination provides sensitive detection of the surface potential and capacitance gradient with nanometer-scale spatial resolution as it was verified on self-assemblies of fluoroalkanes and a metal alloy. The KFM and d*C*/d*Z* applications to several heterogeneous polymer materials demonstrate the compositional mapping of these samples in dry and humid air as well as in organic vapors. In situ imaging in different environments facilitates recognition of the constituents of multi-component polymer systems due to selective swelling of components.

## Introduction

Atomic force microscopy (AFM) applications include high-resolution imaging, probing of local materials properties and compositional mapping of heterogeneous materials in different environments. In recent years the improvements in these fields have been associated with the development of oscillatory modes and multi-frequency approaches. Despite the continuing interest and progress in high-resolution imaging, the practical value of AFM is strongly related to compositional imaging. The high sensitivity of the AFM probe to various materials properties is behind such visualization of individual components of complex materials. So far, compositional imaging of heterogeneous polymer materials is primarily based on differences of local mechanical and adhesive properties of their constituents. These differences are best reflected in phase images in the amplitude modulation (AM) mode, which are obtained at elevated forces. Although the phase contrast is efficient in differentiating the rubbery, glassy and inorganic components of polymer blends and composites, its interpretation in terms of specific mechanical properties is extremely difficult. Furthermore, the quantitative analysis of local mechanical properties of even neat polymers obtained in AFM experiments is rather intricate due to their frequency-dependent nature. An additional limitation of AFM-based nanomechanical studies is their sensitivity to materials with an elastic modulus below 10 GPa (polymers, biological specimen, etc.) that leaves rigid materials (metals, semiconductors, ceramics, etc.) out of reach.

In this situation AFM compositional imaging can be expanded by local electrical techniques that enable measurements of electrical properties (surface potential, dielectric permittivity, capacitance, etc.) at a tip–sample junction. Here we will demonstrate that single-pass Kelvin force microscopy (KFM) studies based on sensing of an electrostatic force gradient can be performed in the intermittent contact mode and provide high-resolution maps of surface potential. This approach will be shortly described and its functionality will be proved by the results obtained on model systems: Self-assemblies of fluoroalkanes and metal alloys. The verification of novel approaches is specifically important in the case of multi-frequency AFM applications that give a researcher a multiple choice of experimental procedures. Furthermore, the initial efforts of compositional imaging using surface potential maps will be demonstrated by studies of individual polymers and polymer blends on different substrates. In some cases we will add complimentary capacitance gradient (d*C*/d*Z*) data that give hints on the local dielectric response of materials.

Finally, we would like to point out that the environmental AFM capabilities, which make this technique unique among the microscopic methods, has enormous potential for compositional mapping of organic materials and polymers. In the intermittent contact operation, proximity of the conducting probe to a sample helps in avoiding screening the sample’s electrical response by a water layer when measurements are performed at high humidity. A selective swelling of individual components with water or organic solvents helps to distinguish them when the experiments are conducted in water and solvent vapors. We will show how environmental studies of polymer blends with AFM-based electric studies enhance compositional imaging of these heterogeneous materials.

## Materials and Methods

### Samples

The samples for KFM and d*C*/d*Z* measurements were prepared by depositing different materials on doped Si, graphite or conducting glass (ITO) substrates. Fluoroalkanes F_14_H_20_ were dissolved in perfluorodecalin and a droplet of its dilute solution (0.01 mg/mL) was spin cast on the substrates. Self-assembled F(CF_2_)_14_(CH_2_)_20_H–F_14_H_20_ structures (toroids, spirals and ribbons) and thin molecular layers were formed on these substrates. A piece of Bi/Sn alloy with a composition 40:60 was squeezed between two flat Si plates at 200 °C and chilled to room temperature. One of the plates was removed afterwards, and a shiny surface of the alloy sheet was examined by AFM. Polymer films were prepared by the spin-casting of a droplet of a dilute solution of the polymer on the substrates. Thin films of poly(methyl methacrylate) (PMMA) and polymer blends PMMA with polystyrene (PS) and PS with poly(vinyl acetate) (PVAC) were prepared from their solutions in toluene. A thin film of a blend of PMMA with poly(vinyledenefluoride) (PVDF) blend was spin cast from its solution in 1-methyl-2-pyrrolidinone. The polymers with molecular weights in the 100–150 K range, solvents and ITO glass substrates were purchased from Sigma-Aldrich. The Bi/Sn alloy was purchased from Rotometals, Inc. We also used boron-doped Si wafers with 0.02–0.05 Ω·cm resistivity manufactured by Virginia Semiconductor, Inc. Fluoroalkane samples were courtesy of Prof. M. Moeller (DWI, Aachen, Germany).

Prior to AFM measurements, scratches were made on the polymer films by a sharp wooden stick and we verified that a substrate-specific morphology was present at the bottom of scratches. At the scratched locations one can measure the film thickness and a relative electrical response of the polymer and Si substrate. All prepared samples were glued to metal disks with epoxy glue. An electrical contact between the instrument and the samples was arranged with a wire, which was fixed to a side of the conducting substrate with a drop of silver glue.

For KFM and d*C*/d*Z* we used Pt-coated Si probes with a stiffness in the 3–40 N/m range and flexural resonance in the 60–300 kHz range. AC and DC voltages were applied to the probe whereas the sample was earthed. The sharper coated probes have a tip diameter around 25–30 nm. Some of the probes were specially made with larger tip size (50–60nm in diameter). In control measurements, we applied carbon nanotubes probes (generously provided by Carbon Design Innovations). The probes with small tip apex and tips with high aspect ratio provide higher spatial resolution of surface potential images whereas the probes with thicker tips have a better signal-to-noise ratio of the surface potential.

The majority of measurements were made in air at 20–25% humidity. An environmental chamber of the microscope was used for studies in humid air (2% < RH < 95%, as measured by a humidity meter) and also for experiments in organic solvent vapors. One or two milliliters of water, methanol or toluene was injected into the environmental chamber, and these liquids gradually evaporated to influence the samples. Because of differences in the boiling points of the liquids, methanol vapor affected a sample in a shortest time, i.e., only a few minutes.

### Electrostatic force measurements

The simultaneous use of the probe flexural resonance frequency (ω_mech_) for sensing van der Waals or mechanical tip–sample interactions for surface profiling and a much lower frequency (ω_elec_) for electrostatic force detection was suggested in 1988 [1]. For many years the single-pass approach has been mostly applied in UHV KFM studies, and such measurements are usually conducted in the non-contact mode. Under ambient conditions KFM is most often applied in the two-pass lift mode [2] that does not require the use of multiple lock-in amplifiers. In the lift mode, the long-range electrostatic force is sensed by a conducting probe, which is positioned 10–20 nm above the sample. This is done in the second pass by guiding the probe along the topography contour determined in the first pass whilst keeping away from the sample. Caution related to possible electrostatic force coupling with topography should still be taken into account. In many cases this method of separating the mechanical and electrostatic forces helps, however, measurements of the electrostatic force at remote tip–sample distances limit their sensitivity and, particularly, spatial resolution. Therefore, it may be advantageous to check the capabilities of single-pass KFM at ambient conditions because nowadays lock-in amplifiers are an essential part of the electronics in scanning probe microscopes.

AFM-based electrostatic force measurements were performed under ambient conditions with an Agilent 5500 scanning probe microscope equipped with a MAC III unit, which has three lock-in amplifiers (LIAs) enabling multi-frequency measurements. The MAC III has three dual phase LIAs converting the AC inputs to amplitude and phase. These digitally-controlled analog LIAs have a broad bandwidth (up to 6 MHz) that cover the operational bandwidth of the photodetector employed in the microscope. A signal access module provides a flexible routing of input and output signals of the LIAs. The software, which is flexible in routing signals back to the controller, supports two servo systems related to these LIAs.

**Figure 1:**
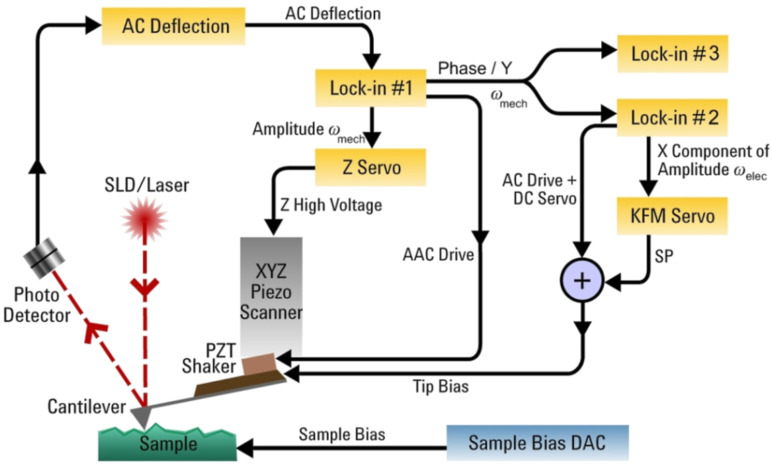
Sketch illustrating implementation of Kelvin force microscopy in the AM–FM mode. Two servo-loops, which are based on the lock-in amplifiers, are employed for the simultaneous detection of the mechanical and electrostatic tip–sample interactions at different frequencies: ω_mech_ and ω_elec_.

The single-pass KFM operation can be realized in different combinations of AM and frequency modulation (FM) modes; AM–AM, AM–FM, FM–FM, FM–AM [3] where first abbreviation defines a surface tracking procedure and the second – detection of the electrostatic force. It is worth noting that AM is associated with force detection and FM with force gradient detection, and this difference appears to be essential for optimization of KFM imaging. The KFM operation can be described with the help of Figure 1. One of LIAs (LIA-1) was used for topography imaging, which was performed at the first flexural resonance of the probes, ω_mech_, with free amplitude *A*_0_ in the 1–100 nm range and set-point amplitude *A*_sp_ = 0.6–0.8 *A*_0_. These imaging conditions correspond to the intermittent contact imaging when *A*_sp_ is chosen on the steep part of the amplitude-versus-distance curve. Another LIA (LIA-2), which is used for KFM, applies AC and DC voltages to the probe and detects the electrostatic response either directly from the photodetector (AM–AM) or from the LIA-1 (AM–FM). The latter block scheme configuration is shown in Figure 1. In the AM–FM, the electrostatic interactions are excited by an AC voltage applied to the probe at ω_elec_ = 3–5 kHz, which is within the bandwidth of ω_mech_. The electrostatic response, which is detected by the phase signal or *Y* component signal of the LIA-1, is seen at the heterodyne frequencies ω_mech_ ± ω_elec_. When the KFM servo is on, the heterodyne sidebands practically disappear and the DC voltage equals the contact potential difference. This AM–FM procedure is similar to one used for KFM in the non-contact regime [4] with the following variations. LIA-1 is used for both the AM topography servo and the demodulation of the side bands of the drive frequency. In [4] a separate PLL was used for the FM topography servo, and the drive out from the FM controller served as the reference input for LIA-1. In our case we used the internal reference of LIA-1 as the drive output to drive the cantilever shaker. There is a noise advantage in the use of the *Y* vector component as the input to the LIA-2 for the KFM servo because this excludes noise from the *X* vector component that would couple in through the phase calculation in the LIA-1.

The third LIA was used for recording the amplitude response at 2ω_elec_ in two different configurations. In one configuration (shown in Figure 1) this amplifier was connected to the LIA-1. In this case the amplitude of 2ω_elec_ satellite of the main amplitude peak at 2ω_mech_ is recorded and it is proportional to the force gradient signal, and therefore to d^2^*C*/d*Z*^2^. In the second configuration, the third LIA is connected directly to the photodetector and in this case the detected amplitude at 2ω_elec_ is proportional to the electrostatic force and therefore to d*C*/d*Z*. Both the d*C*/d*Z* and d^2^*C*/d*Z*^2^ signals are related to the local dielectric permittivity and we used these for compositional mapping. The interplay between the experimental measurements and theoretical studies is needed for a better understanding of the sensitivity of d*C*/d*Z* and d^2^*C/*d*Z*^2^ based dielectric studies, and for the extraction of quantitative permittivity data. In addition, we also recorded the phase response at 2ω_elec_ that can be used for detection of complex dielectric response. In the following we will demonstrate that surface potential and d*C*/d*Z* data, which are measured simultaneously and independently of sample topography, can be used for compositional imaging.

## Results and Discussion

### Studies of model samples

For the verification of our AFM-based electrostatic measurements, we have chosen two model systems: Self-assemblies of fluoroalkanes F(CF_2_)_14_(CH_2_)_20_H–F_14_H_20_ and the binary metal alloy Bi/Sn. The fluoroalkane molecules consist of fluorinated and hydrogenated parts that avoid each other in F_14_H_20_ self-assemblies (spirals, toroids, ribbons) on different substrates [5]. The F_14_H_20_ molecules have a dipole of 3.1 D oriented along the chain at the central –CF_2_–CH_2_-junction. Therefore, macroscopic Kelvin probe studies of Langmuir–Blodgett layers of different F*_n_*H*_m_* revealed a strong surface potential of −0.8 V [6–7] that is assigned to vertically oriented molecular chains with fluorinated parts facing air. Therefore, the fluoroalkane structures are the useful models for the verification of KFM operations. The same is true for metal alloys because their surface potentials are directly defined by work function [8].

**Figure 2:**
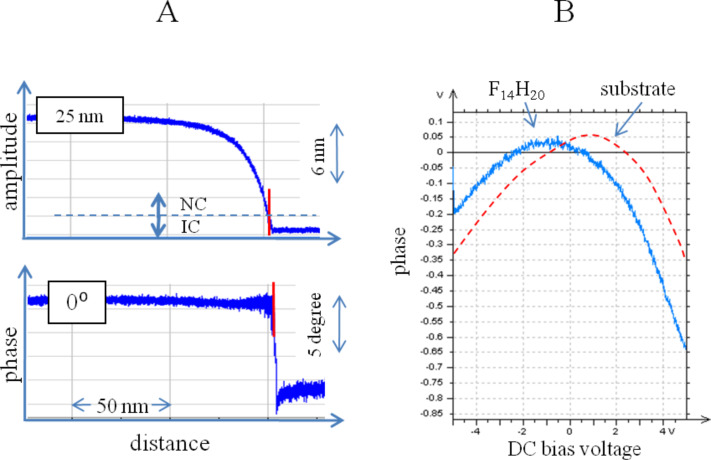
**A** – Graph showing a temporal change of amplitude and phase of the AFM probe on approach to a sample. The time axis is converted to distance axis. The initial value of the amplitude is 25 nm and phase −0 degrees. A red vertical line shows a point where a sharp drop of the phase indicates contact with the sample. On the right side of the line the amplitude was reduced further to stay at the set-point level (*A*_sp_) corresponding to the intermittent contact regime. A horizontal dotted line marks the amplitude set-point level above and below which the non-contact (NC) and intermittent contact (IT) regimes take place, respectively. **B** – Graphs showing the electrostatic force induced phase changes as a function of DC bias voltage. The measurements were made on a sample of F_14_H_20_ on Si substrate with the probe located above the F_14_H_20_ self-assemblies (blue curve) and away from them (red curve).

At the beginning we compare KFM imaging in the non-contact and intermittent contact modes. When the AFM probe, which is driven into an oscillation at its resonant frequency, approaches a sample, the probe amplitude gradually decreases, Figure 2A. This effect is caused by a squeezed air damping and attractive probe-sample force interactions. The latter are enhanced by electrostatic force interactions between the conducting probe and the sample as its counter electrode. The amplitude drop is accompanied by changes of the probe phase. On further approach of the probe to the sample, the amplitude changes are intensified and at some point a sharp drop (4–10 degrees) of the phase is observed. This signifies transition from non-contact situation to the intermittent contact regime. In other words, imaging at the set-point amplitude (*A*_sp_) below its transition value will insure a profiling of surface topography, and at higher *A*_sp_ the imaging will proceed in the non-contact mode when the probe experiences long-range forces such as electrostatic forces. This is illustrated in Figure 2B which shows the dependence of phase changes as a function of DC bias voltage between a conducting probe and different locations of the F_14_H_20_ adsorbate on Si substrate. The phase-versus-DC-bias curve (colored blue) was detected when the probe was over a domain of the toroid-like self-assemblies. It shows a parabolic dependence of the phase response and demonstrates that the electrostatic force is fully compensated (nullified) at the bias voltage (about −1 V) and equals the difference in surface potential of the tip and the sample underneath. Similar phase-versus-DC-bias curve (red colored), which was recorded at a sample location free of the toroids, has been shifted on the DC bias axis due to a different surface potential at this location. The measurement of the phase or frequency responses to DC bias is the subject of electric force microscopy (EFM) whereas the mapping of bias voltages needed for nullification of the electrostatic is the main function of KFM.

**Figure 3:**
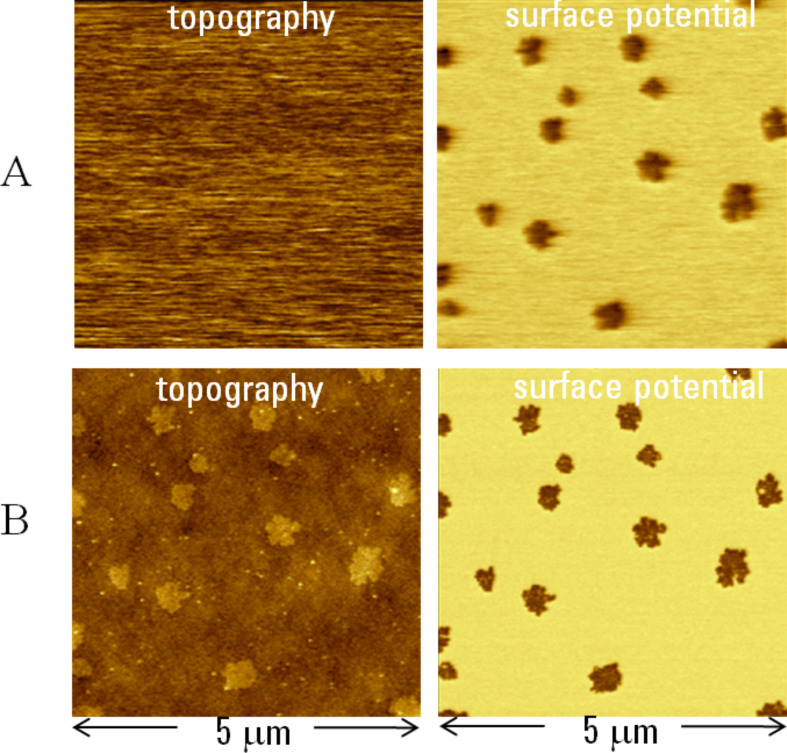
Topography and surface potential images of F_14_H_20_ self-assemblies on Si substrate. The images in **A** were obtained in the non-contact regime with A_sp_ just above the transition level from the non-contact to the intermittent contact regime (see Figure 2A). The images in **B** were recorded in the intermittent contact regime with *A*_sp_ below the transition level. The contrast covers height variations in the 0–10 nm range in the topography images and potential changes in the 0–1 V range in the surface potential images. The averaged surface potential difference between the self-assemblies and the substrate is 0.75 V for the image in **A** and 0.79 V for the image in **B**.

The topography and surface potential images, which were recorded on the F_14_H_20_ adsorbate on Si substrate, are presented in Figure 3. These images were obtained with *A*_sp_ just above (Figure 3A) and below (Figure 3B) its value corresponding to the transition to the intermittent contact regime. The topography image recorded in the non-contact mode is practically featureless. At the conditions near the transition, it may be possible to detect weak cross-talk patterns, which are caused by the long-distance electrostatic interactions that are responsible for the bright domains in the corresponding surface potential images. The topography image changes drastically in the intermittent contact regime and the elevated sub-micron domains of self-assemblies are clearly resolved. Related patterns are detected in the surface potential images obtained in both regimes. The signal-to-noise ratio of the surface potential pattern is higher in the intermittent contact operation due to the larger d*C*/d*Z* amplitude in immediate vicinity of the sample. A higher spatial resolution of the surface potential image obtained in the intermittent contact operation is also obvious. The averaged potential value is slightly larger (−0.79 V vs −0.75 V) in the image obtained in the intermittent contact regime. This statement can be extended to the 2-pass KFM measurements in the lift mode that actually present the results as a combination of the topography image in Figure 3B and surface potential image in Figure 3A.

In our experience the KFM measurements in the intermittent contact studies are most stable and reproducible with *A*_sp_ 70–80% of its value at the initial contact. The lowering of the set-point might cause a tip–sample discharge followed by sample and tip modifications. The fact that under ambient condition, KFM studies in the intermittent contact mode at moderate *A*_sp_ can be performed on pure metals such as Au [9] indicate that a short tip–sample force contact and a airborne contamination of surfaces prevents discharge between the tip and sample.

Here we would like to comment on a comparison of KFM results obtained in the AM–FM and AM–AM modes. The KFM images presented in Figure 3 were obtained in AM–FM mode. The earlier KFM studies of F_14_H_20_ self-assemblies on different substrates revealed that the FM detection of the electrostatic forces provides the most accurate measurements of surface potential (~0.8 V) and higher spatial resolution of surface potential images compared to AM detection [9–11]. This result is consistent with KFM studies of different samples in UHV [4,12] and with the theoretical considerations in [13]. In the latter paper, the authors have reported that compared to the electrostatic force changes, the force gradient variations are more confined to the probe apex and less sensitive to the force contributions of the cantilever and tip body. This hints at the advantage of FM detection compared to AM. We have also performed KFM studies in FM–FM mode using our microscope enhanced by adding PLL control. The surface images of F_14_H_20_ self-assemblies obtained in the FM–FM and AM–FM modes were practically identical when the measurements were performed in the intermittent contact mode. It is also important that the AM–FM and FM–FM measurements are typically performed at smaller stimulating AC voltages than AM–AM ones. This is essential for avoiding the possible electric field-induced changes of surface electric properties.

**Figure 4:**
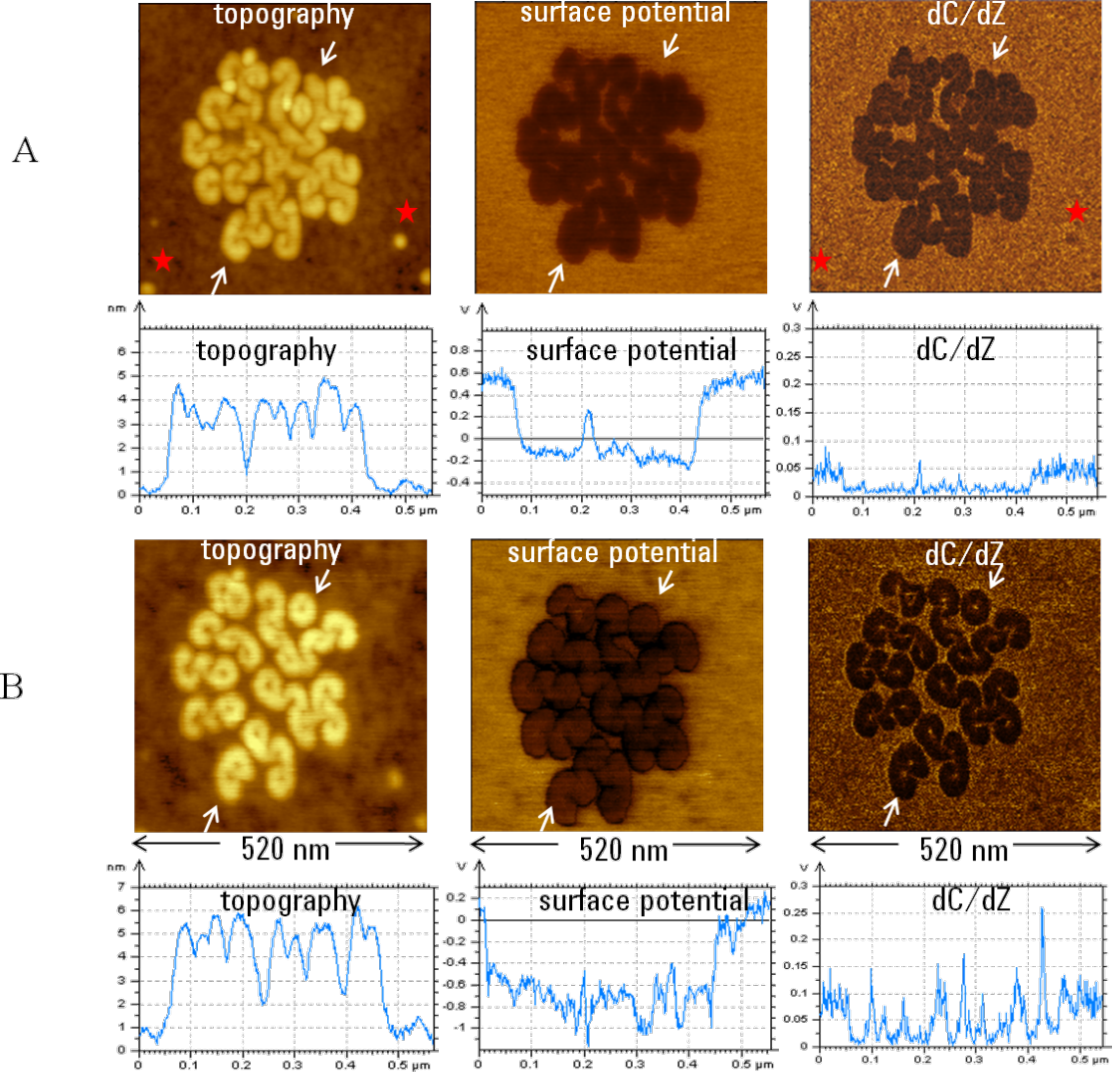
Topography, surface potential and d*C*/d*Z* images and cross-section plots obtained on a domain of F_14_H_20_ self-assemblies on the Si substrate. The plots, which are shown underneath the images, were taken along the directions indicated by the white arrows. The images in **A** were obtained during imaging in air. Two red stars indicate the contaminating particles, which are not seen in the surface potential image. The images in **B** were obtained during imaging in methanol vapor.

The KFM operation in the intermittent contact can be also performed in different gas environments. Importantly, the intermittent contact measurements were not affected by high humidity that screens surface potential when studies are performed in the non-contact mode [14]. Such environmental KFM studies of fluoroalkanes were performed in high humidity [10] and we extended these in a methanol vapor environment. A domain of self-assembled F_14_H_20_ structures is shown in Figure 4A, which demonstrates the topography, surface potential and d*C*/d*Z* images recorded in the single-pass operation. The cross-section profiles taken across the images in the direction marked with white arrows are presented underneath the images. The domain consists mostly of spiral self-assemblies around 4 nm in height as seen from the topography profile. The potential profile shows the negative surface potential of the spirals (approx. −0.8 V), which as mentioned before is caused by an almost vertical orientation of fluoroalkane chains whose fluorinated segments are facing air. The spirals also exhibit a darker d*C*/d*Z* contrast than the surroundings. The latter is formed by a thin fluoroalkane layer with molecules lying along the sample surface that makes them “invisible” in surface potential image. A few contaminating particles, which are marked with the red stars, are seen in the topography and d*C*/d*Z* images but not in surface potential image. The d*C*/d*Z* contrast correlates with variations of dielectric permittivity and the latter is related to averaged dipole values. A quantification of d*C*/d*Z* and permittivity changes is under development, and recent data [15] indicate that d*C*/d*Z* response increases with an increase of sample permittivity. This can explain the more negative d*C*/d*Z* contrast of the spirals. The images, which are shown in Figure 4B and related figures below, were recorded on the sample in methanol vapor. The change of the environment caused a structural transformation of spirals to toroids and the height of these structures increased to ~5 nm. The latter is likely related to straightening of the chain molecules in the vertical direction. This slight change of the molecular alignment might be responsible for the increase of the negative surface potential from −0.8 V to (−1.0)–(−1.1) V. The methanol-induced changes in the d*C*/d*Z* image are responsible for the stronger difference between the contrast of the self-assemblies and the surroundings. Additionally, the toroids centers, a few nm in size, are visible in this image whereas the same toroids are seen as more bulky patterns in the surface potential image. The described height, surface potential and d*C*/d*Z* changes were reversible after the environmental chamber was opened to air. This is not related to the spirals-toroids conversion. There is no doubt that the electrostatic interaction of polar methanol molecules with fluroalkanes is responsible for these changes that initiate the structural transformation and small-scale surface transport on the substrate which is obvious from a comparison of the topography images shown in Figure 4.

The discussed images of F_14_H_20_ adsorbates also illustrate a high spatial resolution of surface potential and d*C*/d*Z* detection in the single-pass operation performed in the intermittent contact mode. A true spatial resolution of KFM is often determined as a width of a transition region between locations of different surface potential [4,12]. In a separate paper [16], we reported the measurements of the potential profile change at the steps of F_14_H_20_ self-assemblies on a Si substrate. When the Pt-coated probe was applied the step width was around 20–30 nm – a dimension that is similar to the tip's apex diameter. The same width was 4–5 nm on imaging with a carbon nanotube probe due to its high aspect ratio. In compositional mapping, the visualization of individual components is more important than obtaining the correct values of local mechanical or electrical properties. Therefore, the spatial resolution can be higher than the described above. In imaging of F_14_H_20_ ribbons on graphite, tiny bright slits of 2 nm in width were distinguished in between the individual ribbons. These are the locations where the probe “feels” the substrate.

**Figure 5:**
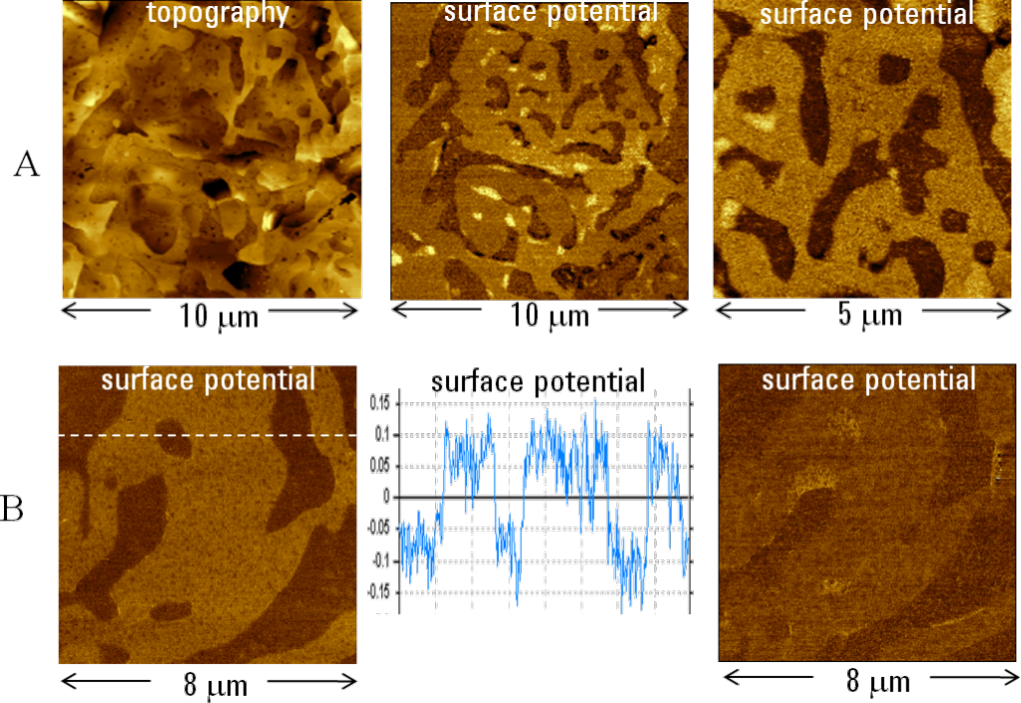
Topography and surface potential images recorded on two Bi/Sn samples. The images in **A** were obtained on the sample that is characterized by several levels of the surface potential contrast. The contrast covers the height corrugations in the 0–60 nm range in the topography image and the potential variations in the 0–0.4 V range in the surface potential images. The surface potential images in **B** were obtained on a freshly-prepared Bi/Sn sample (left) and the same sample after the overnight storage in air (right). The graph between the images displays the potential profile taken in the left image along the direction marked with a dashed white line.

A soldering material, an alloy of Bi and Sn, is another useful sample for KFM studies. The topography and surface potential images of this sample show its surface domain structure presented by different patterns (see Figure 5A). This finding suggests that the material is actually a partial solid solution. A comparison of these images shows that there is no a cross-talk between the topography and potential measurements. The surface potential contrast in the images at different magnifications shows four levels of contrast with the 200 mV span. These changes are close to the difference of surface potentials of Sn and Bi (~0.2 V). We found that sample preparation and its storage are important factors influencing the surface potential contrast. The surface potential image of a freshly prepared sample, Figure 5B (left), shows only binary potential alternation of 0.2 V as seen from the cross-section profile in Figure 5B (middle). Therefore these domains can be assigned to the individual metals Sn and Bi. The surface potential image of the same location after the sample was stored overnight in air is presented in Figure 5B (right). The contrast between the individual domains has worsened and bright patches have appeared in several locations. These changes are most likely caused by oxidation which is more progressive for Sn. This is an example of KFM compositional imaging of a stiff material. Other rigid materials that are beyond the range of phase imaging applications are semiconductors. The KFM inspection of local impurities and defects will benefit from higher-resolution studies in the intermittent contact regime.

### KFM and d*C*/d*Z* mapping of polymer materials

Electrical and dielectric properties of polymer materials are studied by different methods, and their characterization at small scales and in confined geometries is an important and challenging task. The pathway to mapping of d*C*/d*Z* responses of polymers, which are directly related to local dielectric permittivity that depends strongly on frequency, has been initiated by studies of PVAC films [17]. In this work the frequency-dependent d*C*/d*Z* responses of this material, which was previously examined with dielectric spectroscopy [18], were studied with AFM probe at a single location. Furthermore, these studies were extended to d*C*/d*Z* mapping of a PVAC/PS blend at different temperatures in UHV [19] and to studies of a PVAC-based nanocomposite material [20]. We initiated KFM and d*C*/d*Z* measurements of the polymer objects having in mind several objectives. They included, but were not limited to, the use of these methods for compositional imaging of heterogeneous polymers and examination of polymer structures and behavior in different environments. In a wide variety of polymers those with a non-polar nature have a very low dielectric permittivity whilst polar polymer materials have permittivities around 7–9. Many polymers have dipole groups with molecular dipoles oriented along the chain backbone or perpendicular to it. In addition, the polymer response to an AC electric field is described by complex dielectric permittivity directly related with a spectrum of molecular motions and its dependence on temperature. Therefore the development of AFM-based electric techniques capable of examining these materials on a sub-micron scale and in a wide frequency range will open up a broad range of technologically and fundamentally important applications.

**Figure 6:**
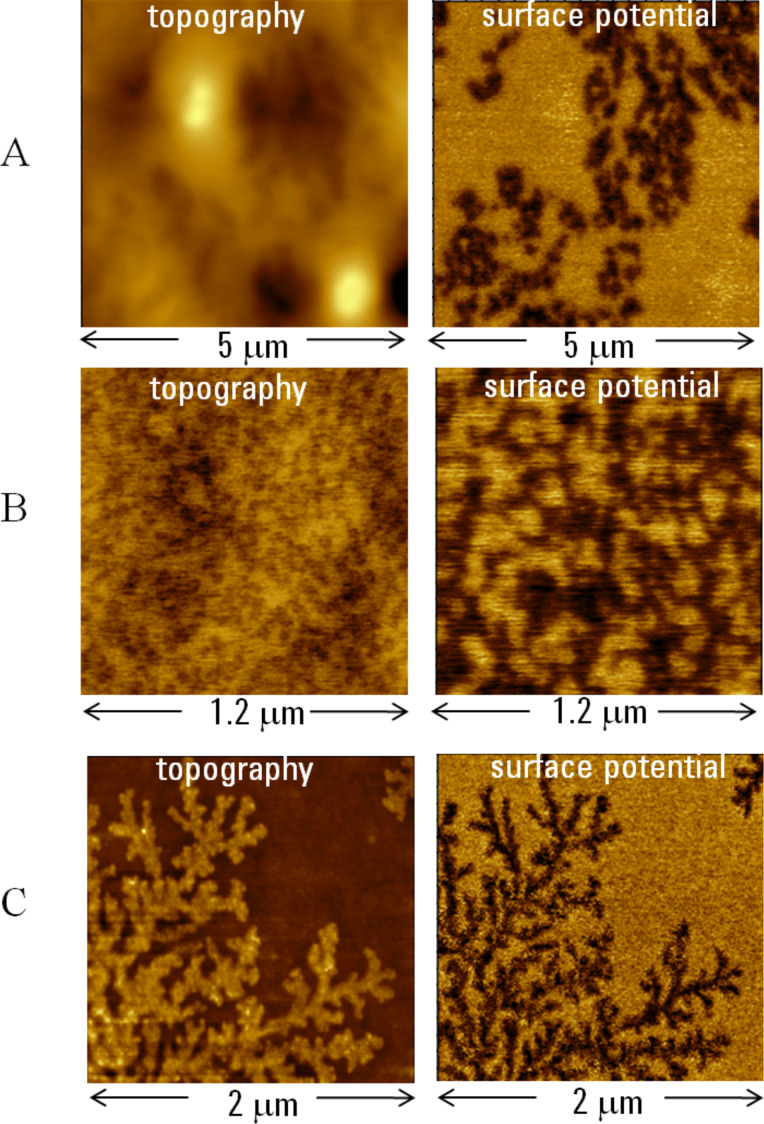
Topography and surface potential images of the films of a latex blend of poly(*n*-butyl acrylate) and poly(pentafluorostyrene) are presented in **A** and **B**. The contrast covers height variations in the 0–140 nm and 0–10 nm ranges in the topography images and potential changes in the 0–0.4 V and 0–1V ranges in the surface potential images. Topography and surface potential images of a film of a PMMA and PVDF blend are shown in **C**. The contrast covers the height corrugations in the 0–10 nm range in the topography image and the potential variations in the 0–1 V range in the surface potential image.

The first example is taken from studies of a binary latex blend of poly(*n*-butyl acrylate) and poly(pentafluorostyrene). The images of the blend film at two locations are shown in Figure 6A and Figure 6B. The surface potential images reveal a bi-component composition of this material: The darker locations can most likely be assigned to the fluorinated component. The surface potential contrast between the constituents was relatively strong around 0.4 V. The micro-phase separated morphology is more homogeneous in the second location. It is worth noting that due to the softness of this polymer material, the images were recorded at a much lower *A*_sp_ at which the tip was partially imbedded in the sample. Therefore, the surface potential measurements can be carried out even in sub-surface layers. Another example of the materials with a fluorinated component is a thin film of a PMMA and PVDF blend. The relation between the polymer morphology and material performance is the key question for polymer technology that reinforces the importance of compositional imaging.

The film was prepared by spin-casting of PMMA and PVDF solutions, and the blend was formed during evaporation of the solvent and crystallization of PVDF. Therefore, dendritic structures observed on film surface represent crystalline PVDF. The dark surface potential contrast of the structures is consistent with the presence of a molecular dipole (~2.1 V) in this polymer and the preferential orientation of these dipoles is in the vertical direction. In crystalline polymer materials with a strong dipole moment, KFM might be a useful tool for correlating the molecular dipole orientation with chain orientation in lamellar structures.

**Figure 7:**
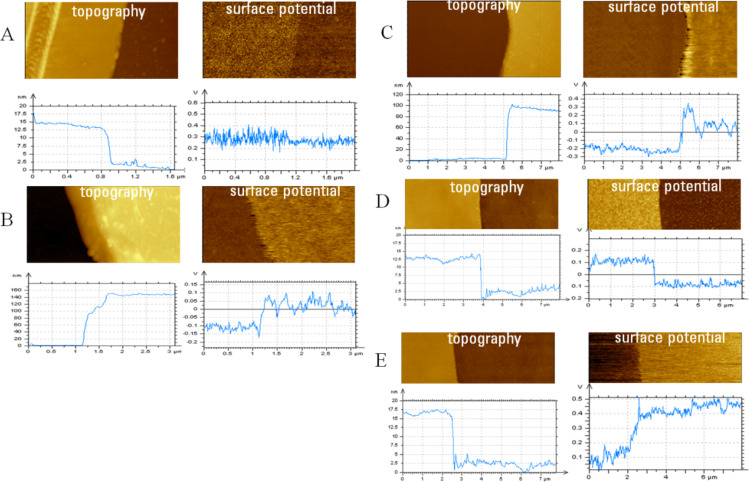
Topography and surface potential images, which were recorded at the scratch location in PS films of different thicknesses on a Si substrate, are presented in A and B. Similar images at the scratch location in PMMA films on Si are shown in **C**–**E**. The cross-section profiles, which were taken in the images, in the horizontal direction are shown under the images. The images in **A**–**D** were recorded with the sample in air and the image in **E** was recorded with the PMMA film in methanol vapor.

In materials with strong dipole moments, the surface potential is directly related to the strength and orientation of the molecular dipole, as is the case in fluoroalkanes self-assemblies. In other materials surface potential correlates to the surface work function of metals, the doping level of semiconductors, the strength and orientation of molecular dipoles, and the presence of charges and interfacial and field-induced dipoles. In polar polymers, the situation can be much more complicated and the apparent surface potential of polymer molecules has to be discussed in connection with macroscopic Kelvin probe studies of thin PMMA films. These studies revealed that surface potential of PMMA films depends on the stereoregularity and molecular conformations of this polymer [21]. Therefore, PMMA domains and blocks in multi-component polymer materials might exhibit a specific surface potential that can be examined with KFM. First of all, we studied the surface potential variations between PMMA and PS films of different thickness on a Si substrate. For this purpose, the images were collected in the scratched regions of the polymers with different thickness, Figure 7A. The cross-section profiles of the topography and surface potential images revealed that compared to the Si substrate, the surface potential of PS is rather small (~50 mV) in a film of thickness ~12 nm and twice as high in a film which is 140 nm thick. The surface potential difference between a 100 nm thick PMMA film and Si reaches 300 mV. Monitoring of the environmental effects was demonstrated by following the topography and surface potential changes of a thin PMMA film, Figure 7B. The study of swelling of PMMA film by different organic vapors [22] showed that methanol has a strong effect as shown by AFM. Indeed, swelling of PMMA with methanol induced changes not only in topography but also in surface potential. The surface potential changes are relative and the contrast of the film and the substrate reversed on sample exposure to methanol vapor: The difference of 200 mV between the PMMA film and Si became ca. −300 mV. These alterations proceed within 30–40 min, as the methanol vapor spread throughout the chamber and modified the sample. After the chamber was opened to air, the reverse changes of surface potential contrast happened practically immediately following methanol evaporation. This suggests that the methanol vapor affects only the top surface of the sample.

The analysis of KFM data obtained under ambient conditions in the intermittent contact mode on different samples shows that absolute values of surface potential might be influenced by a presence of occasional contaminants or modifications of the tip and the sample. This should be taken into account in comparing the surface potential data in Figure 7A and Figure 7B. A quantitative difference of surface potentials at dissimilar surface locations or sample components obtained in the same image is more reliable than absolute potential values.

**Figure 8:**
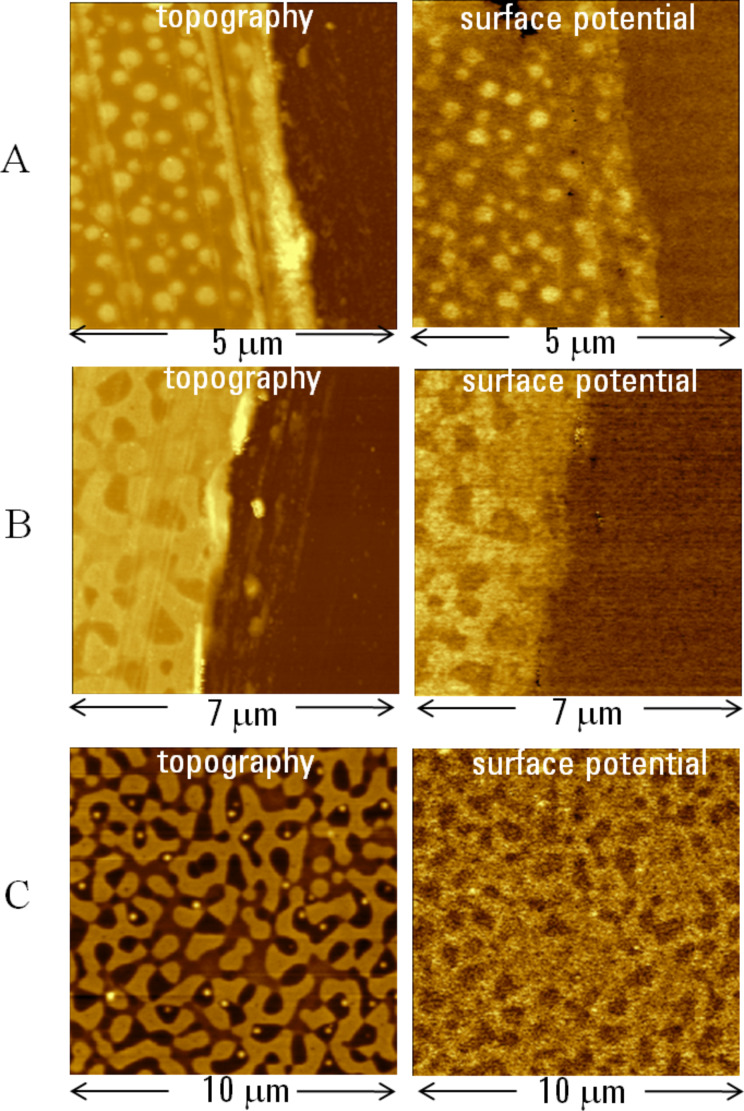
Topography and surface potential images of films of PS/PMMA blends on a Si substrate. The images in **A** and **B** were obtained in air on a scratch location in 70% PS–30% PMMA and 30% PS–70% PMMA blends, respectively. The images in **C** were obtained on surface of 30% PS–70% PMMA blend in humid air (RH = 95%) after the sample had spent two days in this environment. The contrast covers the height corrugations in the 0–55 nm, 0–75 nm and 0–20 nm ranges in the topography images in **A**–**C** and the potential variations in the 0–1.1 V, 0–0.7 V and 0–0.6 V ranges in the surface potential images in **A**–**C**.

KFM studies of PMMA and PS films were further extended by imaging of their blends with weight ratios of 3:7 (3M7S) and 7:3 (7M3S). Their topography and surface potential images, which were recorded at the scratches, are shown in Figure 8. According to the cross-section of the topography images, the films have a thickness of around 30 nm and the surface corrugations due to the elevated domains are in the 4–7 nm range. The averaged potential differences between the bright locations of the blends and the substrate were around 300 mV for 3M7S and 170 mV for 7M3S. On the blends’ surface the differences between the brighter and darker locations were around 150–200 mV (3M7S) and 80 mV (7M3S). As can be seen, the surface potential patterns of the blends resemble their topography. However, a surface potential pattern with reversed contrast appeared when an AC bias was applied to the sample and not to the tip. The surface potential contrast of the blends was best seen at an AC bias of 6 V when the noise was much lower than at an AC bias of 1 V. In case of the 3M7S blend, as the AC bias was changed from 3 V to 6 V a substantial increase (80 mV to 150 mV) in surface potential difference between the bright and dark locations was observed. This might be considered as an indication of field-induced dipole effect.

The allocation of the surface potential features to the blend constituents is not a trivial task and a direct correlation with the results obtained on PS and PMMA films might be difficult due to unknown morphology inside the blend film. We assume that the bright and dark patches correspond to domains enriched in PMMA and PS. This assignment is tentatively supported by a correlation of the ratio of bright and dark areas to the composition of the blends as well as by the higher surface potential values recorded on PMMA films compared those with PS. Decisive support for this analysis was found in the images of the 7M3S blend obtained after the sample was exposed to high humidity (RH > 95%) overnight (Figure 8C). The surface potential image was unchanged but multiple droplets appeared inside the dimples in the topography image. Most likely these are due to condensed water droplets on hydrophobic surface of PS-enriched domains. The droplets are characterized by a relatively large wetting angle that indicates hydrophobicity of the underlying locations.

Compared to surface potential studies the use of d*C*/d*Z* measurements was relatively limited by studies of organic layers [23] and water adsorption [24–25]. Recently, the situation has changed and there is now an increasing interest in nanoscale dielectric studies. Our interest in PS-PVAC blends was brought about by recent efforts to measure its local dielectric properties by different EFM approaches [17,19–20,26]. The static dielectric permittivities of the blend components are quite different (2–3 for PS and ~7 for PVAC) as well as their dipole moments (~0.3 D for PS and 2.1 D for PVAC). This makes this material attractive for local electric measurements. In addition, the glass transition temperature of PVAC is quite low (35 °C) thus its complex permittivity can also be studied with comparative ease. The studies EFM-based local measurements [19] were conducted at frequencies in the range 0.1–100 Hz range and at temperatures around 35 °C (glass transition of PVAC) where the dielectric response exhibits pronounced changes. Indeed, it was found that the dielectric contrast of the PVAC domains varied with temperature, and nanoscale mapping of the permittivity differences was demonstrated. These studies were done in UHV and the d*C*/d*Z* measurements were conducted in the non-contact mode.

**Figure 9:**
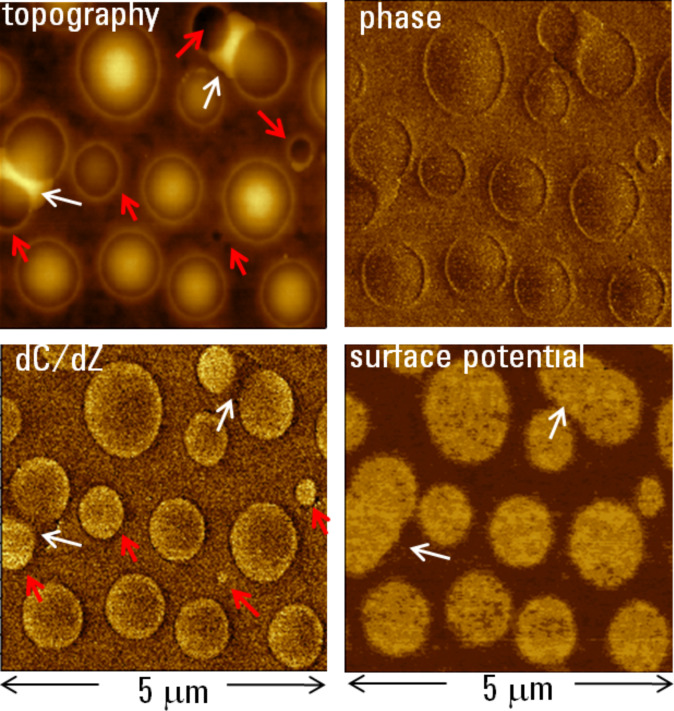
Topography, phase, surface potential and d*C*/d*Z* images of an 80 nm thick film of PVAC/PS blend on ITO glass. The contrast covers height corrugations in the 0–45 nm range, phase changes in the 0–45 degrees range, surface potential variations in 0–0.4 V range and d*C*/d*Z* alternations in the 0–80 mV range.

In extending single-pass KFM and d*C*/d*Z* applications in the intermittent contact, we examined 80-nm thick film of the same blend on an ITO substrate. The topography, phase, d*C*/d*Z* and surface potential images of one of the locations are shown in Figure 9. The topography image revealed a morphology, which was characterized by sub-micron scale domains embedded into a matrix. The domains have a shape of a top part of sphere inserting into the surroundings. The elevated part of highest domains reached 30 nm where as few of domains are seen below the matrix. This morphology is similar to that described in [19] where the round-shape domains were assigned to PVAC and the matrix to PS. The topography image also shows well-resolved rims around the PVAC domains that most likely are a consequence of immiscibility of the components of this blend. The composition map of the blend is clearly presented in the surface potential image in which PVAC domains exhibit a 50–60 mV higher surface potential than the PS matrix. Their potential is also 130–140 mV higher than that of the ITO substrate as seen in surface potential image taken at the scratch in the film (not shown here). Remarkably, in a few surface regions the neighboring PVAC domains are connected by “bridges”, which are marked with white arrows. According to the surface potential contrast, these bridges are formed from PVAC. The surface potential contrast reflects the larger dipole moment of PVAC and the positive value is caused by an average dipole orientation towards the substrate. In further speculation, we might point out that because the dipole moment of PVAC is oriented perpendicular to the molecular chain [27], a planar chain orientation could be the most preferable arrangement in the PVAC domains. High-resolution surface potential images (not shown here) emphasize that surface potential contrast is not uniform across the PVAC domains. This observation suggests a clustering of polymer chains into nanometer-scale blocks with different averaged molecular orientation.

The phase and d*C*/d*Z* images are quite different from that for surface potential. The phase image resembles the error signal image and the PVAC domains are not emphasized. At room temperature both polymers, PS and PVAC, are in glassy state therefore it would not be expected to observe a difference in their phase contrast. The d*C*/d*Z* contrast variations are more distinguished with PVAC domains being brighter than the surrounding PS. There are two interesting aspects. The first is related to the contrast of individual domains. The domains, which are marked with a red arrow, have their top part just above the surface or below it. The d*C*/d*Z* patterns of these domains are uniform in bright contrast. The other elevated domains exhibit a different d*C*/d*Z* patterns with a central part darker that the perimeter. This suggests that at elevated locations only the tip apex is sensing the electrostatic force and a large part of the tip participates in the force interactions when the domains are lower. The second is related to the bridges between the PVAC domains (white arrows). The darker d*C*/d*Z* contrast as opposed to brighter surface potential contrast points to their assignment as PVAC. We might suggest that these bridges are formed by ultrathin PVAC films spreading between the two domains and that the d*C*/d*Z* contrast is more influenced by the underlying PS matrix than the surface potential contrast. This is only a tentative suggestion but it indicates the necessity of knowing the depth of the surface potential and d*C*/d*Z* measurements.

**Figure 10:**
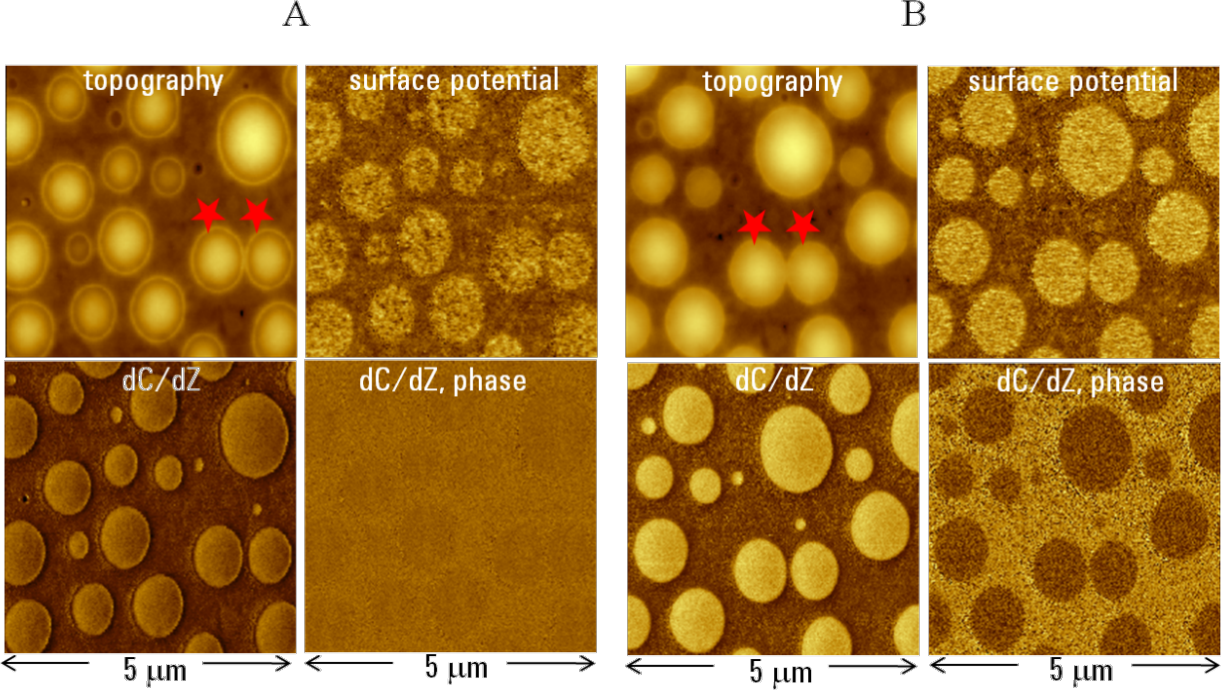
Topography, surface potential and d*C*/d*Z* (amplitude and phase) images of 80 nm thick film of PVAC/PS blend on ITO glass. The images in **A** were obtained in air and the images in **B** in high humidity (RH = 95%). The contrast covers height corrugations in the 0–35 nm range, surface potential variations in 0–0.6 V range, d*C*/d*Z* amplitude alternations in the 0–180 mV range and d*C*/d*Z* phase changes in the 0–20 degrees range in both sets of the images.

The studies of the PVAC/PS blend were continued at low and high humidity and also in methanol and toluene vapors. There are general similarities between the results obtained in high humidity and in the organic vapors. The images of the same location obtained in air and at high humidity are shown in Figure 10. The images obtained in air exhibit similar features to those seen in Figure 9. In addition to the topography, surface potential and d*C*/d*Z* amplitude signal, we were able to demonstrate the d*C*/d*Z* phase image, which does not show much contrast in air. By changing air to humid environment, we expected selective action on the hydrophilic PVAC domains. The images, which were obtained after the sample was exposed high humidity for couple of hours, are presented in Figure 10B. The two red star marks placed near the same PVAC domains serve as the references in Figure 10A and Figure 10B. The humidity effect is pronounced in the topography and d*C*/d*Z* (amplitude and phase) images. Surface potential changes are less obvious. A selective swelling of PVAC domains with water vapor led to an increase in the volume of the domains and the disappearance of the circular rims. The height of the domains marked with the red stars increased from 20 to 25 nm. Simultaneously with the topography changes, the d*C*/d*Z* contrast increased 8-fold and a pronounced phase contrast (~20 degrees) was detected. These changes were reversible and the original contrast of all three images was restored after the environmental chamber was opened or purged with argon. The strong phase changes might serve as an indication of dynamic dielectric behavior that is common for polymers around glass transition point. The swelling of polymers with low-molecular agents effectively lowers their glass transition point and this effect is suspected. Ongoing d*C*/d*Z* studies of this blend at elevated temperatures will help address this question.

The d*C*/d*Z* contrast observed in the PVAC/PS blend and its environmental changes are not easy to understand. The static permittivity of PVAC is much higher than that of PS but it is difficult to assume that the contrast recorded at 3 kHz follows their low frequency difference. We also might suspect some environmental effects in our preliminary measurements at low humidity (3% RH) and in different gases (N_2_, Ar) as revealed by the the contrast variations. The humidity-induced changes are very noticeable and well as those caused by methanol and toluene vapors. These observations might be also affected by dielectric absorbance of water or organic molecules that, being in GHz range, might also have direct or indirect lower frequency contributions. Therefore, the expansion of AFM-based d*C*/d*Z* measurements to broader (higher and lower) frequency ranges is quite desirable for a better understanding the local dynamic dielectric properties.

## Conclusion

Single-pass KFM and d*C*/d*Z* studies in the intermittent contact regime were carried out and their value was verified in experiments with two model samples, i.e., self-assemblies of fluoroalkanes F_14_H_20_ on a Si substrate and films of the metal alloy Bi/Sn. The electrostatic force interactions were measured by force gradient changes. The results showed that sensitivity and spatial resolution of this approach is superior compared to use of the non-contact mode for KFM and d*C*/d*Z* detection. Furthermore, the single-pass measurements of several polymer materials demonstrate that KFM and d*C*/d*Z* mapping can be applied for compositional imaging of multi-component systems. These techniques have also been applied to samples in various environments (humidity, vapors of organic solvents, etc.), where the samples were subjected to partial swelling. Such measurements are helpful in the identification of individual constituents of complex materials and will further enhance compositional imaging. The d*C*/d*Z* measurements, which were performed at a single frequency, gave rise to a number of intriguing questions regarding the origin of the image contrast. Expansion of these studies to a broad frequency range and at different temperatures will be essential for reliable interpretation of the dielectric data and will be the subject of nanoscale dielectric spectroscopy.
